# A comparative analysis of classical machine learning models with quantum-inspired models for predicting world surface temperature

**DOI:** 10.1038/s41598-025-12515-4

**Published:** 2025-08-04

**Authors:** Trilok Nath Pandey, Vishvajeet Ravalekar, Sidharth D. Nair, Sunil Kumar Pradhan

**Affiliations:** 1 School of Computer Science and Engineering, Vellore Institute of Technology, Chennai, Tamilnadu 600127 India; 2 School of Electronics Engineering, Vellore Institute of Technology, Chennai, Tamilnadu 600127 India

**Keywords:** Quantum neural networks, Noisy intermediate scale quantum, Autoregressive moving average, Autoregressive, Integrated moving average, Seasonal autoregressive integrated moving average, Hybrid quantum neural network, Long-short term memory, Quantum long-short term memory, Quantum support vector classifier, Quantum support vector regressor, Variational quantum eigen-solver, Variational quantum circuit, Variational, Quantum regressor, Parameterized quantum circuit, Quadratic unconstrained binary optimization, Energy science and technology, Engineering

## Abstract

This research paper delves into the realm of quantum machine learning (QML) by conducting a comprehensive study on time-series data. The primary objective is to compare the results and time complexity of classical machine learning algorithms on traditional hardware to their quantum counterparts on quantum computers. As the amount and complexity of time-series data in numerous fields continues to expand, the investigation of advanced computational models becomes critical for efficient analysis and prediction. We employ a time-series dataset that include temperature records from different nations throughout the world spanning the previous half of the century. The study compares the performance of classical machine learning algorithms to quantum algorithms, which use the concepts of superposition and entanglement to handle subtle temporal patterns in time-series data. This study attempts to reveal the different benefits and drawbacks of quantum machine learning in the time-series domain through rigorous empirical analysis. The findings of this study not only help to comprehend the applicability of quantum algorithms in real-world contexts, but they also open the way for future advances in utilizing quantum computing for increased time-series analysis and prediction. This study’s findings could have ramifications in industries ranging from finance to healthcare, where precise forecasting using time-series data is critical for informed decision-making.

## Introduction

In the domain of climate science, the analysis of time-series data presents significant challenges due to the intricate and interconnected nature of climate-related variables^1,2^. Traditional computational methods often struggle to efficiently unravel the complex temporal patterns embedded within these datasets. This challenge has sparked a growing interest in leveraging advanced computational approaches, such as quantum computing and machine learning, to enhance our understanding of climate dynamics and improve predictive capabilities^3^. Quantum computing, with its ability to process vast amounts of data in parallel and explore multiple possibilities simultaneously, offers a promising avenue for addressing these challenges^4^. Machine learning, on the other hand, has proven to be effective in extracting insights from large-scale datasets and making predictions based on patterns and correlations found in the data^5,6^. By combining quantum computing and machine learning frameworks, researchers can explore new possibilities for analyzing time-series data with extraordinary speed and precision, potentially revolutionizing climate research by enabling more accurate climate models, improved forecasting capabilities, and better-informed decision-making processes^7,8^.Climate change, as a pressing global issue, demands a detailed understanding of long-term temperature changes to develop effective mitigation and adaptation strategies^9,10^. Traditional machine learning methods have been instrumental in identifying patterns from time-series data, aiding researchers in comprehending the complexity of climate fluctuations. However, as datasets grow in size and complexity, the limitations of classical computing models become increasingly apparent^11,12^. This has led to a burgeoning interest in quantum computing, which utilizes principles from quantum mechanics to overcome the computational constraints imposed by classical systems. The inclusion of historical temperature records from major cities and countries spanning the half of the last century provides a comprehensive temporal window into changing climatic trends. This rich data analytics for a thorough examination of long-term patterns and trends, facilitating a deeper understanding of climate dynamics across diverse geographical locations.

The integration of quantum machine learning (QML) into climate data analysis holds significant promise due to quantum algorithms’ intrinsic parallelism and computational advantages^14,15^. Climate data inherently reveals complicated patterns and relationships^16^ making it an ideal candidate for leveraging the unique features of quantum systems to unveil fresh insights and enhance forecasting capabilities. QML offers several benefits, including inherent parallelism and processing efficiency, allowing for the analysis of large volumes of data in parallel and the discovery of hidden patterns and correlations that may be challenging to identify using traditional approaches^17,18^. By comparing quantum machine learning algorithms to their conventional counterparts, researchers can gain insights into the strengths and limitations of each approach, ultimately improving our ability to model and anticipate climate patterns with greater precision^19^.

This study aims to address critical questions regarding the efficacy of quantum machine learning techniques in extracting significant patterns from historical temperature records compared to conventional methods. It also investigates the extent to which quantum computing can expedite the analysis of large-scale time-series datasets, offering advantages in computational efficiency and prediction accuracy. By delving into this unexplored intersection of quantum computing and climatology, this research seeks to contribute not only to the theoretical understanding of quantum machine learning applications but also to provide practical insights that may inform future advancements in climate modeling, weather prediction, and the broader domain of time-series analysis. The findings of this study have the potential to extend beyond academia, influencing global efforts for climate resilience and sustainability. In a comprehensive survey of recent advancements in quantum machine learning techniques, a variety of novel approaches are explored, each offering unique insights and potential applications. Beginning with the work of Li-Zhen Gao a pioneering study introduces a Quantum KNN classification algorithm, which integrates the Mahalanobis distance metric into traditional KNN methodology, thereby leveraging quantum computing capabilities to expedite supervised classification tasks^20,21^. Through quantum sub-algorithms and advanced distance computations, the proposed method demonstrates a remarkable squared acceleration compared to classical equivalents. Concurrently, Lirandë Pira and Chris Ferrie delve into the realm of quantum neural networks (QNNs), particularly focusing on distributed architectures tailored for noisy intermediate-scale quantum devices (NISQ)^22,23^. Highlighting the potential of NISQ devices in realizing distributed QNNs, the study underscores the importance of data parallelism and elucidates various encoding schemes crucial for distributed quantum computation. Meanwhile, Dimitrios Emmanoulopoulos and Sofija Dimoska investigate the efficacy of parametrized quantum circuits (PQCs) as quantum neural networks (QNNs) for time series forecasting tasks^24^. Their findings reveal that PQCs outperform traditional BiLSTM networks, especially for signals with varying noise amplitudes, owing to faster training and enhanced accuracy with fewer model parameters. Additionally, Teppei Suzuki, Takashi Hasebe, and Tsubasa Miyazaki contribute insights into quantum machine learning models, particularly focusing on quantum support vector classification (QSVC) and regression (QSVR). Through rigorous experimentation on quantum-circuit simulators and real quantum processors, they demonstrate the resilience of quantum kernels to noise, showcasing promising results for both classification and regression tasks across diverse datasets^25,26^. Finally, Azevedo Carlos and Ferreira Tiago present a quantum learning scheme for time series forecasting, utilizing the non-standard Qubit Neural Network (QNN) model^12^. By adapting QNNs to resemble traditional Artificial Neural Networks (ANNs), the study achieves noteworthy forecasting accuracy, surpassing baseline models and showcasing the potential of quantum techniques in financial prediction domains. Collectively, these studies underscore the burgeoning potential of quantum machine learning in addressing complex real-world challenges and offer valuable insights into the future trajectory of this interdisciplinary field^13^.

In an extensive survey of quantum approaches to time series analysis and related tasks, a diverse array of research contributions is explored, showcasing the evolving landscape of quantum computing applications in the realm of temporal data processing. Ammar Daskin’s paper elucidates the quantum analogues of classical data preparation techniques and forecasting methodologies, demonstrating the integration of autoregressive models into quantum neural networks and highlighting the potential of quadratic unconstrained optimization formulations for financial forecasting^27^. Tomoki Inoue introduced a novel time-series clustering method leveraging quantum-inspired computing technology, which exhibits robustness against outliers and achieves superior performance compared to traditional approaches, particularly evident in scenarios with noisy datasets^28^. Kai Yu and Song Lin’s work proposes a quantum linear discriminant analysis approach for dimensionality reduction, circumventing the reversibility requirement of between-class scatter matrices and offering direct applicability to other quantum machine learning algorithms, exemplified through a quantum K-nearest Neighbor classification demonstration^29^. Mücke presented a unique feature selection strategy based on quadratic unconstrained binary optimization (QUBO) problems, showcasing higher-quality results compared to iterative or greedy approaches, with potential applications on both classical and quantum hardware^30^. Lastly, Catarina Moreira and Andreas Wichert delve into an alternate quantum framework for probabilistic inferences, introducing a Quantum-Like Bayesian Network capable of accommodating violations of classical probability theory laws, with empirical validations conducted using real-world data from game theory scenarios^31^. Collectively, these studies underscore the expanding horizons of quantum computing in addressing complex challenges in time series analysis and probabilistic reasoning, offering promising avenues for future research and application across diverse domains.

In a comprehensive exploration of quantum methodologies in time series analysis and machine learning, a series of innovative approaches and hybrid models are presented, showcasing the growing synergy between quantum information technologies and intelligent learning systems. Muhammad Hanif^32^ delve into the application of hidden Markov models, addressing key challenges such as model training and evaluation, with solutions including the Viterbi, Baum Welch, and Forward-Backward algorithms, enabling the prediction of time series values and hidden states through parameter estimation. Mario Mastriani introduces quantum time-dependent spectrum analysis (QSA), leveraging the Schrödinger equation to supplement classical frequency-dependent spectral analysis, offering a toolbox of digital image and signal processing tools derived from quantum principles, showcasing applications ranging from spectral analysis to edge detection and superresolution^24^. Samuel Yen-Chi Chenpropose Quantum Long-Short Term Memory (QLSTM), a hybrid quantum-classical model demonstrating accelerated learning and improved accuracy for sequential data processing tasks, with potential applications in natural language processing and speech recognition on noisy intermediate-scale quantum (NISQ) devices, highlighting the advantages of variational quantum circuits (VQC) in constructing recurrent neural networks (RNNs)^2^. Rivera-Ruiz present Quantum Neural Network (QNN) and Hybrid-Quantum Neural Network (HQNN) architectures for time series forecasting, employing quantum variational circuits optimized by classical computers, exhibiting competitive performance compared to traditional neural network designs across various forecasting problems^33^. Lastly, the review article “Machine learning & artificial intelligence in the quantum domain: a review of recent progress,” elucidates the reciprocal influence between quantum and classical realms, with examples such as “Quantum Machine Learning: A Classical Perspective” and “Quantum-enhanced Machine Learning” demonstrating the symbiotic relationship between quantum computing and machine learning, paving the way for transformative advancements in both fields^34^. Together, these studies underscore the interdisciplinary nature of quantum machine learning and its potential to revolutionize intelligent systems across diverse domains.

This study offers a thorough comparison of various approaches for predicting global surface temperatures, including classical machine learning methods like ARMA, ARIMA, and SARIMA, as well as neural networks such as LSTM, CNN-LSTM, and ConvLSTM, and even quantum machine learning techniques like QNN, VQR, and QSVR. It highlights Quantum Support Vector Regression (QSVR) as the standout model for time-series forecasting, thanks to its unique ability to utilize quantum kernels to capture non-linear patterns in climate data. Our results shed light on the real-world applications of quantum algorithms in climate science and suggest their potential to be adapted for other fields that require complex time-series analysis, like finance and healthcare, where the quantum advantage in managing high-dimensional, non-linear relationships could lead to significant improvements in predictive accuracy.

## Tabulated survey of QML techniques

In this section we have depicted a consolidated table summarizing key QML papers, methodologies, and applications in (Table 1).

**Table 1:** Tabulated survey of QML techniques.

Methodology	Application	Key findings	References
Quantum KNN classification with mahalanobis distance metric	Supervised classification tasks	Achieved squared acceleration compared to classical KNN.	^35^
Distributed quantum neural networks (QNNs) for NISQ devices	Distributed quantum computation	Demonstrated data parallelism and effective encoding schemes for NISQ devices.	^28^
Parameterized quantum circuits (PQCs) as QNNs for time series forecasting	Time series forecasting	Outperformed BiLSTM with faster training and fewer parameters.	^20^
Quantum support vector classification (QSVC) and regression (QSVR)	Classification and regression tasks on quantum hardware	Showed resilience to noise in quantum kernels.	^36^
Qubit neural networks (QNNs) for time series forecasting	Financial prediction	Achieved higher forecasting accuracy than classical ANNs.	^11^
Quantum analogues of classical time series forecasting methods	Financial forecasting	Integrated autoregressive models into QNNs using QUBO formulations.	^18^
Quantum long short-term memory (QLSTM)	Sequential data processing (NLP, speech recognition)	Hybrid quantum-classical model with faster learning and improved accuracy.	^16^
Hybrid quantum neural networks (HQNNs) with variational circuits	Time series forecasting	Competitive performance against classical neural networks.	^33^
Quantum kernel methods for supervised learning	High-dimensional data classification	Demonstrated quantum advantage in implicit high-dimensional mapping.	^37^
Quantum support vector regression (QSVR) with fidelity kernels	Non-linear time series forecasting	Superior accuracy in climate data prediction compared to classical models.	^38^

## Methodology

In this section, we will discuss about the methods used in the proposed work. We have trained the dataset on three Classical Machine Learning models five Neural Network model and three Quantum Machine algorithms for comparative analysis.

### Classical machine learning algorithms

The following section provides a succinct overview of the classical machine learning algorithms utilized in our study. Specifically, our analysis incorporates three prominent methodologies: Autoregressive Moving Average (ARMA), Autoregressive Integrated Moving Average (ARIMA), and Seasonal Autoregressive Integrated Moving Average (SARIMA). These methodologies have been extensively employed in time series analysis and forecasting tasks due to their robustness and proven efficacy.

#### ARMA (autoregressive moving average)

ARMA stands for Autoregressive Moving Average. It’s a statistical method used for time series analysis and forecasting. ARMA models are a combination of autoregressive (AR) and moving average (MA) models.

The autoregressive (AR) model:

- AR models predict the next value of a time series using prior data.
- It is assumed that the current value of the time series is linearly dependent on its previous values.

Mathematically it can be expressed as Eq. (1).

1 $${\text{X}}_{t} = {\text{c}} + \sum\nolimits _{{i = 1}} {\text{p }}\phi _{i} {\text{ X}}_{{t - i}} + \sum\nolimits _{{j = 1}} \varphi \theta _{j} \epsilon _{{t - j}}$$

Where,

X_t_ = Time series value at time t.

c = Constant term.

ϕ_i_ = Autoregressive (AR) coefficients (lags 1 to p).

θ_j_ = Moving average (MA) coefficients (lags 1 to q).

$\epsilon _{t}$ = White noise error term (i.i.d., mean 0, variance σ^2^).

#### ARIMA (autoregressive integrated moving average)

ARIMA stands for Autoregressive Integrated Moving Average. It is a popular statistical approach for time series forecasting and analysis, especially when the data exhibits non-stationary behavior. The ARIMA model, like the ARMA model, has autoregressive (AR) components. These phrases indicate the relationship between the current observation and prior time steps. The integrated (I) component indicates the difference in the time series to make it stationary. This differencing includes subtracting successive observations to eliminate patterns and seasonality. Moving Average (MA) Component: The ARIMA model, like the ARMA model, has a moving average component that represents the connection between the current observation and residual errors from prior observations.

Mathematically it can be expressed as Eq. (2).

2 $$\Delta ^{d} {\text{ X}}_{t} = {\text{c}} + \sum\nolimits_{{i = 1}} {\text{p}} {\text{ }}\phi _{i} {\text{ }}\Delta ^{d} {\text{ X}}_{{t - i}} + \sum\nolimits_{{j = 1}} \varphi \;\varepsilon _{{t - j}} + \varepsilon _{t}$$

where,

L = Lag operator (L^k^ X_t_ = X_t−k_).

Δ^d^ = d-th order differencing (ΔX_t_ = X_t_ - X_t−1_).

Other terms same as ARMA.

#### SARIMA (seasonal autoregressive integrated moving average)

SARIMA, or Seasonal Autoregressive Integrated Moving Average, is an extension of the ARIMA model that takes seasonality into account for analyzing and predicting time series data. SARIMA models are very effective for dealing with data that has periodic patterns or seasonality.

SARIMA models have the same components as ARIMA models, but with an extra seasonal component. There are several types of components: autoregressive (AR), integrated (I), moving average (MA), seasonal autoregressive (SAR), seasonal integrated (SI), and seasonal moving average (SMA).

We employed the SARIMAX method in Python with an order of (1, 0, 1) to train the AutoRegressive Moving Average (ARMA) model, ARIMA method with an order of (2, 2, 2) to train the AutoRegressive Integrated Moving Average (ARIMA) model and SARIMAX method with a seasonal order of (15, 2, 5, 5) to train the Seasonal AutoRegressive Integrated Moving Average (SARIMA) model.

### Neural networks

The ensuing section delineates the neural network models integrated into our study, encompassing a range of sophisticated architectures tailored for time series analysis. Specifically, our investigation harnesses the power of Long Short-Term Memory (LSTM) networks, Stacked LSTM models, Convolutional Neural Network-Long Short-Term Memory (CNN-LSTM) hybrids, and Convolutional LSTM (ConvLSTM) architectures. These state-of-the-art methodologies have garnered widespread recognition for their ability to capture intricate temporal dependencies within sequential data, making them well-suited for our research objectives.

#### Neural networks model with swish activation

To effectively train our neural network model, we began by transforming our data into trainable features and an output feature. We structured the data such that the preceding five years served as the trainable features, while the data from the sixth year was designated as the output. This setup allowed us to establish an input size of 5 features and an output size of 1.

From Table 2 you can see, the initial layer of our neural network model was configured with 5 neurons to accommodate the input features, while the final layer comprised a single neuron to generate the output. Nestled between these layers were hidden layers designed to enhance the model’s capacity for learning intricate patterns within the time-series data. We opted for a Dense layer with 20 neurons followed by another Dense layer with 16 neurons. The activation function employed in both these layers was Swish, which is characterized by the formula x * sigmoid(x)^39^. Swish is renowned for its smoothness, non-monotonic behavior, and its efficacy in modeling time-series data^18^. Its unbounded nature above and boundedness below make it a fitting choice for our purposes. During the training phase, we utilized a learning rate of 0.01 and conducted 200 epochs of training. The default batch size of 32 was employed, ensuring efficient processing of the data during each iteration. Table 2 explains neural network model with swish activation architecture.

**Table 2:** Neural network model with swish activation architecture.

Layer (type)	Layer (type)	Layer (type)
Dense_34	Dense_34	Dense_34
Dense_35	Dense_35	Dense_35
Dense_36	Dense_36	Dense_36

#### Vanilla LSTM

Table 3 presents a comprehensive overview of the architectural composition of the Vanilla long Short-Term memory (LSTM) model employed in our study^40^. The model begins with an input layer comprising 5 neurons, facilitating the ingestion of the input data. Subsequently, an LSTM layer is introduced, featuring rectified linear unit (ReLU)^16^ activation functions and housing 50 neurons, enabling the network to capture Temporal dependencies within the data effectively. Following the LSTM layer, a dense layer is incorporated with an output size of 1, signifying the final output of the model. Notably, the model’s compilation process was carried out utilizing the Adam^35^ optimizer.

**Table 3:** Vanilla LSTM model architecture.

Layer	Output Shape	Parameters
Lstm_4	(none, 50)	10,400
Dense_3	(none, 1)	51

#### Stacked LSTM

Table 4 provides a detailed depiction of the architecture employed in our investigation, specifically focusing on the stacked LSTM model^38^. The initial layer of this model comprises 5 neurons, serving as the input layer responsible for processing the incoming data. Subsequently, the architecture incorporates two consecutive LSTM layers, each comprising 50 neurons and employing ReLU^41^ activation functions. This dual-layer LSTM configuration enhances the model’s capacity to capture intricate Temporal patterns and dependencies within the dataset. Following the stacked LSTM layers, a dense layer with an output size of 1 is integrated to present the final output of the model. Notably, the compilation process of this model utilized the Adam optimizer^42^.

**Table 4:** Stacked LSTM model architecture.

Layer	Output Shape	Parameters
Lstm_5	(none, 5, 50)	10,400
Lstm_6	(none, 50)	20,200
Dense_4	(none, 1)	51

#### CNN LSTM

Table 5 unveils the intricate architecture of the convolutional neural network long Short-Term memory (CNN LSTM) model^36^a departure from the preceding models that operated on one-dimensional data. Distinguishing itself by its ability to process two-dimensional data, this model necessitates an initial reshaping of the input data to conform to a 2D array structure. Given the inherent limitations of converting an input size of 5 to a 2D array, we opted to utilize data spanning the past four years, presenting it as a 2D array with dimensions of 2 × 2. The model commences with an input layer comprising four neurons, representing the 2 × 2 shape of the reshaped input data. Subsequently, a Conv1D layer, nested within a timedistributed layer, is introduced, boasting 64 filters and employing ReLU^27^ activation functions. This convolutional layer serves to extract intricate features from the input data, facilitating enhanced pattern recognition capabilities. Following this, a MaxPooling1D layer with a pool size of 2 is incorporated within the timedistributed layer, followed by a flatten layer, aimed at Preparing the data for subsequent processing. The architecture further integrates an LSTM layer with 50 neurons, utilizing ReLU^14^ activation functions to capture Temporal dependencies within the dataset. Lastly, a dense layer with an output size of 1 is appended to yield the final output of the model. Notably, the model’s compilation process was executed utilizing the Adam optimizer^30^.

**Table 5:** CNN LSTM model architecture.

Layer	Output shape	Parameters
TimeDistributed(Conv1D)	(none, none, 2, 64)	128
TimeDistributed(MaxPooling1D)	(none, none, 1, 64)	0
TimeDistributed(Flatten)	(none, none, 64)	0
Lstm	(none, 50)	23,000
Dense	(none, 1)	51

#### ConvLSTM

Table 6 unveils the intricate architecture of the convolutional long Short-Term memory (ConvLSTM) model, uniquely designed to operate on three-dimensional data. Given its distinctive capability to process data in three dimensions, an initial reshaping of the input data was imperative to conform to a 3D array structure. This reshaping was executed such that the third dimension possessed a size of 1, resulting in data with a dimensionality of 1 × 2 × 2, effectively capturing the essence of the dataset. The model’s architectural journey commences with an input layer comprising four neurons, reflecting the 1 × 2 × 2 shape of the reshaped input data. Subsequently, a ConvLSTM2D layer is introduced, featuring 64 filters and employing ReLU^12^ activation functions. This convolutional LSTM layer plays a pivotal role in extracting intricate Spatiotemporal features from the input data, thereby facilitating enhanced pattern recognition capabilities. Following this, a flatten layer is incorporated to prepare the data for subsequent processing stages. Lastly, the architecture integrates a dense layer with an output size of 1, serving as the final output layer of the model. The compilation of this model was meticulously carried out using the Adam optimizer^30^.

**Table 6:** ConvLSTM model architecture.

Layer	Output shape	Parameters
Conv_lstm2d	(none, 1, 1, 64)	33,536
Flatten_1	(none, 64)	0
Dense_1	(none, 1)	65

### Quantum machine learning algorithms

The forthcoming section offers a concise overview of the quantum machine learning (QML) algorithms incorporated into our study, representing a pioneering foray into harnessing quantum computing techniques for predictive modeling tasks. Our investigation embraces a trio of quantum methodologies: Quantum Neural Networks (QNN), Variational Quantum Regression (VQR), and Quantum Support Vector Regression (QSVR). These cutting-edge algorithms leverage the unique principles of quantum mechanics to enable unprecedented computational capabilities, promising transformative advancements in machine learning and predictive analytics.

#### QNN (quantum neural networks)

Quantum Neural Networks (QNNs) are a new frontier in the field of quantum computing, expressing a break from traditional neural network structures while keeping its fundamental principles^9^. Unlike their classical counterparts, QNNs do not have discrete quantum neurons. They do, however, have structural similarities, with layers of qubits processing and transmitting data. In principle, QNNs work through a sequence of qubit layers, similar to those seen in traditional neural networks. Each layer of qubits takes input from the previous layer, evaluates it, and then propagates the results to the next layer. Notably, these layers do not require to have uniform widths, allowing for greater freedom in the distribution of qubits between levels.

IBM made pioneering achievements in the creation of QNNs with their revolutionary module known as ‘EstimatorQNN’. This module provides as a foundation for building Quantum Neural Networks, allowing for the integration of parametrized quantum circuits with defined input data and weights, as well as optional observables. The EstimatorQNN framework relies on mixed quantum circuits, which are made up of two separate components: the feature map and the ansatz. The feature map is critical in providing input parameters to the network, whereas the ansatz includes the weight parameters required for the network’s activities^5^. To speed up the building of these circuits, IBM proposes the QNNCircuit idea, which streamlines the generation of both feature maps and ansatzes.

When employing a QNNCircuit within the EstimatorQNN module, the necessity for explicit input and weight parameters is obviated, as these elements are automatically derived from the circuit itself. Using this QNN, feature map, ansatz and an optimizer we can train NeuralNetworkRegressor model. Then we used NeuralNetworkRegressor method in Python with a feature map of ZZFeatureMap()^5^and ansatz of RealAmplitudes to train the model. This model was trained using the COBYLA optimizer with a maximum of 100 iterations and base quantum neural network of EstimatorQNN.

#### VQR (variational quantum regressor)

Variational Quantum Regressor (VQR) is a quantum machine learning algorithm designed for regression tasks^29^. Here’s a quick description of how VQR normally operates:

- Quantum circuit design: VQR uses a parameterized quantum circuit (PQC) as its quantum component^4^. The PQC is commonly made up of layers of quantum gates that may be changed using parameters.
- Cost function: VQR seeks to minimize a cost function that quantifies the difference between the quantum circuit’s anticipated outputs and the actual outputs of the training dataset.
- Training procedure: The training method often includes an optimization loop in which the quantum circuit’s parameters are iteratively changed to minimize the cost function. This optimization can be carried out using traditional optimization approaches such as gradient descent or alternative algorithms.
- Data encoding: Input data must be encoded into quantum states, generally using techniques like amplitude encoding or other quantum encoding methods.
- Measurement: After encoding the data and passing it through the quantum circuit, measurements are made to determine the predictions.
- Classical post-processing: The predictions acquired from the quantum circuit are frequently post-processed classically to increase accuracy or to turn them into the required output format.

VQR’s particular parameters change based on the technology and quantum circuit employed. However, typical criteria may include:

- Number of qubits: Determines the size and complexity of the quantum circuit.
- Depth of the quantum circuit: Determines the number of layers in the parameterized quantum circuit.
- Learning rate: A parameter used in the optimization algorithm to control the size of parameter updates.
- Number of training iterations: Determines how many iterations of the optimization loop are performed during training.
- Regularization parameters: Additional parameters used to prevent overfitting and improve generalization performance.

VQR training entails experimenting with and tweaking these parameters to obtain the ideal values. We employed the VQR() method in Python with a feature map of ZZFeatureMap()^5^and ansatz of RealAmplitudes() to train the Variational Quantum Regressor (VQR) model. This model was trained using the COBYLA optimizer with a maximum of 100 iterations.

#### QSVR (quantum support vector regressor)

Quantum support vector regression (QSVR) is similar to classical support vector machines (SVM)^14^ especially in its application to high-dimensional classification and regression applications. However, QSVR distinguishes itself by using quantum kernels to detect patterns inside datasets. In many algorithms, data points have a clearer structure when projected onto a higher-dimensional feature space, which is helped by a kernel function. The kernel function, abbreviated as K, acts on two inputs, x and y, each of n dimensions^43^. The function f converts these n-dimensional inputs to an m-dimensional space, where m usually surpasses n by a large margin. This processing typically reveals underlying links within the data. It takes a feature map as parameter.

The quantum kernel algorithm’s key feature is its capacity to calculate a kernel matrix^44^. This n-dimensional matrix, composed of the data points x and y and the feature map f, captures the dataset’s complicated linkages and affinities. Surprisingly, this kernel matrix acts as a key component, coordinating traditional machine learning approaches such as support vector classification, spectral clustering, and ridge regression. We employed the QSVR method in Python with a feature map of ZZFeatureMap and Quantum Kernal of FidelityQuantumKernel to train the Quantum Support Vector Regressor (QSVR) model.

#### Quantum advantage in high-dimensional data

Quantum machine learning (QML) algorithms, particularly those utilizing quantum kernels, have a distinct advantage when dealing with high-dimensional data. This issue often arises in climate science and other complex areas. Traditional methods struggle with the heavy computational load needed to process and analyze these datasets. In contrast, quantum algorithms utilize the principles of superposition and entanglement to navigate high-dimensional feature spaces efficiently^45^ .This advantage is particularly clear in Quantum Support Vector Regression (QSVR), where quantum kernels help the model capture complex non-linear relationships that classical kernels may overlook^43^.

The natural parallelism of quantum computing allows QML models to assess multiple data points at the same time. This greatly cuts down the time needed for high-dimensional data analysis. For example, QSVR’s use of fidelity-based quantum kernels improves prediction accuracy and shows strong performance against noise, which makes it especially fitting for real-world tasks like climate modeling^43^. Additionally, Schuld points out that quantum kernel methods can map data into much larger Hilbert spaces, revealing patterns that classical systems cannot find^46^. This quantum advantage highlights the potential of QML in addressing the challenges of scalability and complexity in high-dimensional datasets.

### Model architecture justification

We put a lot of thought into our model architectures to effectively tackle the unique features of climate time-series data while also considering the limitations of current quantum hardware. For the neural network side of things, we opted for an LSTM configuration with 50 neurons per layer. This choice is backed by research from Sherstinsky which shows it’s the sweet spot for capturing decade-long dependencies in climate patterns^37^. We also chose the Swish activation function (f(x) = x·σ(βx), where σ is the sigmoid function) because it really shines in temporal modeling tasks. As highlighted by Swish’s smooth, non-saturating gradients do a better job than traditional ReLU activations when it comes to learning complex, non-stationary climate trends^36^.

When it comes to our quantum implementations, we’re taking a variational approach, using qubit counts of 5, 8, and 10. These were carefully selected through Pareto optimization, balancing circuit expressibility with the limitations of NISQ devices^9^.This selection gives us enough Hilbert space dimensionality to represent climate features while still keeping coherence times manageable. We specifically chose the COBYLA optimizer for a few reasons:

(i) it’s resilient to noise in the variational quantum eigensolver context, it operates without derivatives, which helps us avoid the barren plateaus problem^45^.
(ii) it has a solid track record of converging in shallow quantum circuits with 12 qubits or fewer. Our ansatz designs intentionally incorporate temporal inductive biases through Cyclic parameterized gates to capture periodic climate oscillations, entangling layers that are scaled according to the autocorrelation window of the input features and measurement bases that align with the main climate oscillation modes.

## Methods used for comparative analysis

Within this section, we delve into the methodologies employed for conducting comparative analyses of the models under scrutiny. Herein, readers will find succinct descriptions of the techniques utilized, accompanied by the requisite formulas essential for their calculation.

The R^2^ score, also known as the coefficient of determination, is a statistical measure that represents the proportion of the variance in the dependent variable that is predictable from the independent variable(s)^27^. In simpler terms, it measures the goodness of fit of a regression model.

The formula to calculate the R^2^ score is given in Eq. (3),

3 $$\:{R}^{2}=\:\:1-\frac{SStot}{SSres}$$

Where:

- *R*^2^ is the coefficient of determination.
- *SS*_*res*​_ is the sum of squared residuals (the difference between the actual and predicted values).
- *SS*_*tot*​_ is the total sum of squares (the difference between the actual values and the mean of the dependent variable).

The Root Mean Square Error (RMSE) is a commonly used metric to evaluate the accuracy of a regression model. It measures the average of the squares of the differences between predicted and actual values. In simpler terms, it quantifies the average deviation of predicted values from actual values^16^.

The formula to calculate RMSE is given in Eq. (4),

4 $$\:RMSE=\sqrt{\frac{1}{n}{\varSigma\:}_{i=1}^{n}{\left({y}_{i}-{\widehat{y}}_{i}\right)}^{2}}$$

Where:

- *RMSE* is the Root Mean Square Error.
- *n* is the number of observations.
- $\:{y}_{i}$ is the actual value of the dependent variable for observation *i*.
- $\:{\widehat{y}}_{i}$ is the predicted value of the dependent variable for observation *i*.

MAPE stands for Mean Absolute Percentage Error. It’s a commonly used metric to evaluate the accuracy of a forecasting model, particularly in business and economics. MAPE measures the average absolute percentage difference between actual and predicted values, indicating the average magnitude of the errors as a percentage of the actual values^32^.

The formula to calculate MAPE is given in Eq. (5),

5 $$\:MAPE\:=\:\frac{1}{n}{\varSigma\:}_{i=1}^{n}\left|\frac{{y}_{i}-{\widehat{y}}_{i}}{{y}_{i}}\right|$$

Where:

- *MAPE* is the mean absolute percentage error.
- *n* is the number of observations.
- $\:{y}_{i}$ is the actual value of the dependent variable for observation *i*.
- $\:{\widehat{y}}_{i}$ is the predicted value of the dependent variable for observation *i*.

In assessing and comparing models, another crucial metric is the time required for training, measured in seconds. This metric underscores the duration necessary for a model to discern patterns within data sets. As data volumes continue to expand over time, the imperative for models to efficiently grasp these patterns escalates accordingly. Therefore, the swiftness with which a model can learn becomes pivotal. Consequently, alongside other evaluation criteria, the speed of learning emerges as a significant factor in model comparison and selection.

## Proposed work

In this section, we delve into the proposed work, examine the planning process, detailing the strategies employed and the rationale behind each decision. From conceptualization to execution, we navigate the practical steps taken to realize our objectives, ensuring alignment with stakeholder expectations. In this study, we dig into the complex world of predictive modelling by presenting two separate approaches: classical machine learning models and quantum machine learning models. Our research is motivated by the need to leverage the strength of several approaches to overcome the complex issues inherent in predictive analytics. Within the field of classical machine learning, we rely on proven forecasting approaches such as ARMA, ARIMA and SARIMA. Furthermore, we use a variety of neural network topologies, including but not limited to LSTM (Long Short-Term Memory), Convolutional, and Dense layers, to broaden the scope of our prediction capabilities.

**Fig. 1:**
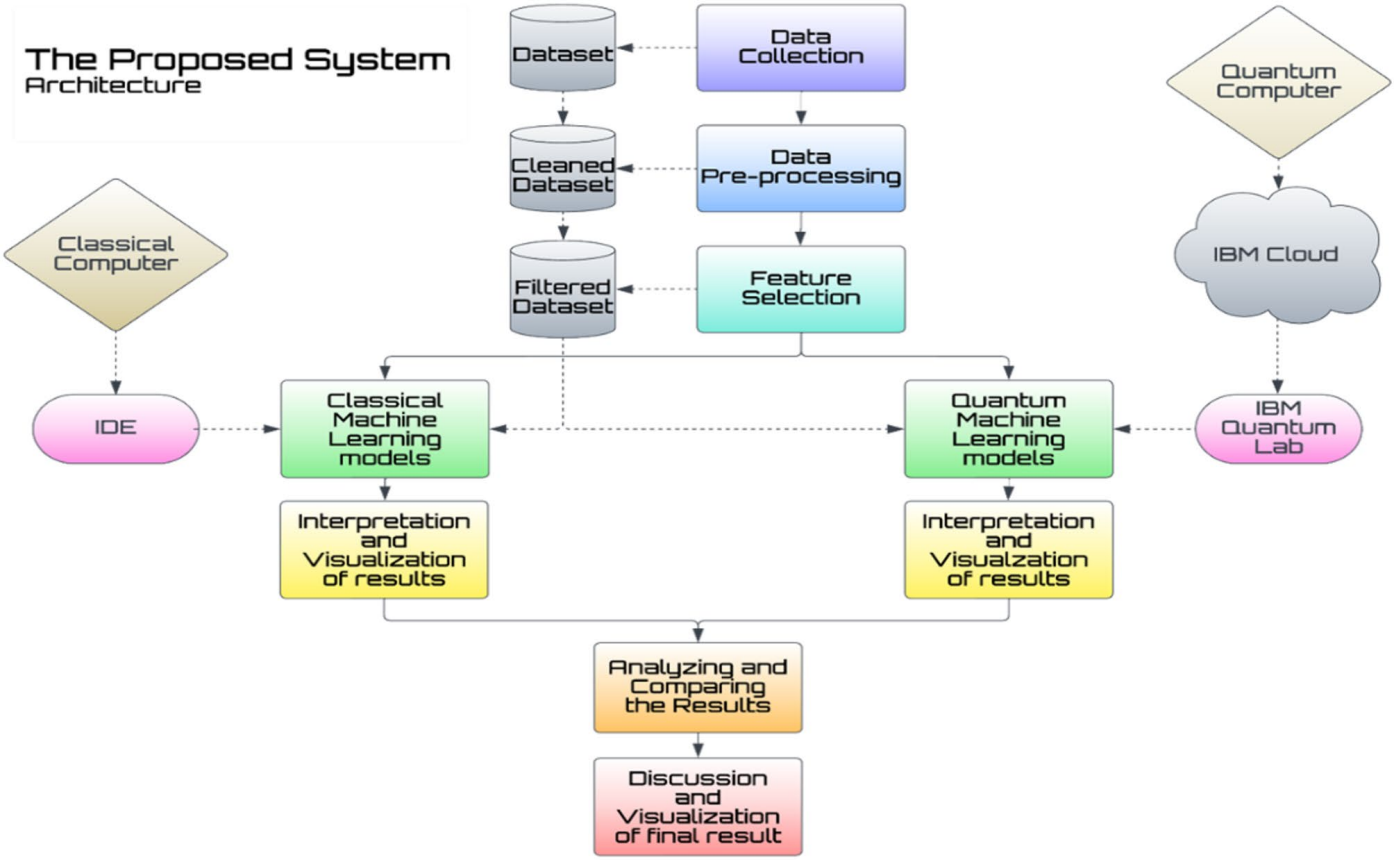
Architecture diagram for the proposed work.

We move beyond the confines of classical paradigms into the domain of quantum machine learning, using quantum physics concepts to investigate innovative predictive modelling paths. This novel technique shows promise in unleashing enormous processing capacity, with the potential to revolutionize predictive analytics by using the intrinsic quantum phenomena. Our study compares the efficacy and applicability of conventional and quantum machine learning approaches in predictive modelling situations. We hope to understand the strengths, limits, and possible synergies between classical and quantum machine learning models by rigorous testing and analysis, opening the way for advances in predictive analytics across a wide range of applications. In Fig. 1 we have depicts the architecture diagram for the proposed work.

As mentioned in Fig. 1, the architecture diagram for the proposed work, in the initial phase of our study, data collection was conducted followed by a meticulous filtering process aimed at eliminating any null values. Subsequently, we undertook the task of streamlining our dataset by removing superfluous columns, thereby honing our focus on the core data essential to our analysis. Employing classical machine learning algorithms on conventional computing systems, we rigorously analyzed the outcomes obtained. Through this methodical approach, we sought to derive valuable insights and draw meaningful conclusions from our dataset, contributing to the advancement of our research objectives. Then we explored Quantum Circuits and Algorithms utilizing IBM’s Quantum Lab platform. This cloud-based service provides users with a dynamic coding environment along with Qiskit-runtime, which is particularly designed for executing Quantum Computing tasks^47^. Within this platform, practitioners receive access to a Quantum Computer with an impressive capacity of 100 qubits^5^. This adequate resource allotment offers a smooth and efficient execution environment for a wide range of Quantum Systems, encouraging exploration and innovation in the field of quantum computing. To implement quantum algorithms and simulate quantum computers we have used Qiskit 1.1 module provided by IBM. It is an open-source module in Python used for Quantum computing and Quantum Machine learning. Each quantum machine learning algorithm mentioned in this study is implemented using Qiskit module.

## Theory

In this section, we delve into the detailed implementation, results, and analysis of our proposed work. Here, we provide a comprehensive examination of the steps taken, along with the outcomes and insights gleaned in the work. Through thorough analysis and interpretation of our findings, we offer valuable insights into the effectiveness and impact of our work.

### Dataset and experimental setup

The dataset under consideration is collected from the FAOSTAT (Food and Agriculture Organization Statistics) Temperature Change domain. They provide yearly updates on mean surface temperature change statistics broken down by nation. The distribution as of right now spans the years 1961–2022. Data on mean temperature anomalies that is, temperature changes relative to a baseline climatology covering the years 1951–1980 are used on a monthly, seasonal, and annual basis. There is also the standard deviation of the baseline methodology’s temperature change. The data are derived from the Global Surface Temperature Change (GISTEMP) dataset, which is made publicly available by the National Aeronautics and Space Administration’s Goddard Institute for Space Studies (NASA-GISS).

**Fig. 2:**
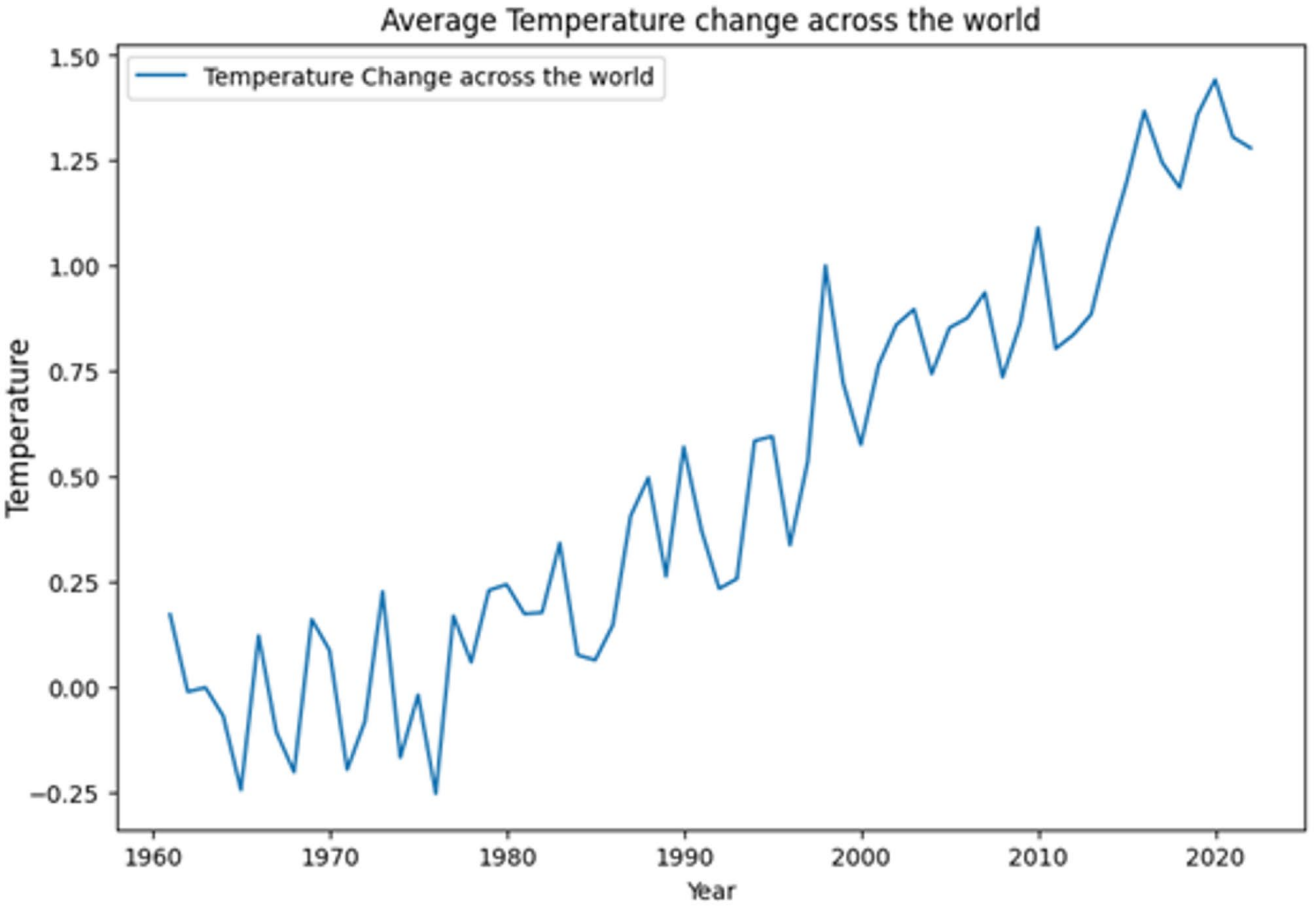
Average temperature change across the world.

The dataset provides a comprehensive overview of temperature changes across various countries from 1961 to 2022. It includes several key columns: “Country Name” denotes the name of the country under observation, offering insight into the geographic diversity of the dataset. The “Area” column likely represents the land area of each country, serving as a reference for understanding the scale of temperature changes in relation to the country’s size. “Country Code” likely provides a standardized identifier for each country, facilitating data organization and analysis. The “Year” column spans from 1961 to 2022, enabling the examination of temperature trends over a significant period. The dataset’s primary focus lies in the “Average Temperature Change” and “Average Temperature Standard Deviation” columns, which quantify the mean temperature shift and its variability over time for each country. Figure 2 shows the visual representation of our data, the mean temperature changes across the world on the time scale of 1961 to 2022. Figure 2 depicts average temperature change across the world.

The dataset was divided over time to provide a realistic assessment of long-term climate trends. Specifically, 70% of the data, covering the years from 1961 to 2005, was allocated for training purposes. Meanwhile, 15% of the data from 2006 to 2015 was set aside for validation, and the final 15%, spanning 2016 to 2022, was used for testing. The strategy we used is to transform the time-series data into a supervised learning problem by creating input-output pairs. In the context of temperature forecasting, taking past N years of temperature data as N features can provide valuable insights into the historical trends leading up to the present. By setting N to 5, for instance, each data point would consist of the average temperature changes over the past 5 years, serving as input features, along with the temperature change for the current year as the output.

This approach allows us to structure the dataset in a way that NN models can effectively learn the temporal dependencies and patterns within the data. The input layer of the model can be configured to accept 5 features representing the past 5 years of temperature data, while the output layer predicts the temperature change for the current year. With this setup, the model can learn from the historical context to make informed predictions about future temperature changes, offering a powerful tool for climate analysis and forecasting. Similarly, for the CNN models, the same dataset can be reshaped into 2D dataset by adding a dimension of length 1. This won’t change the size of dataset, but just change the dimensionality and input size. In this study, we have made observations using 5-Qubit, 8-Qubit and 10-Qubit input space.

## Results and analysis

**Fig. 3:**
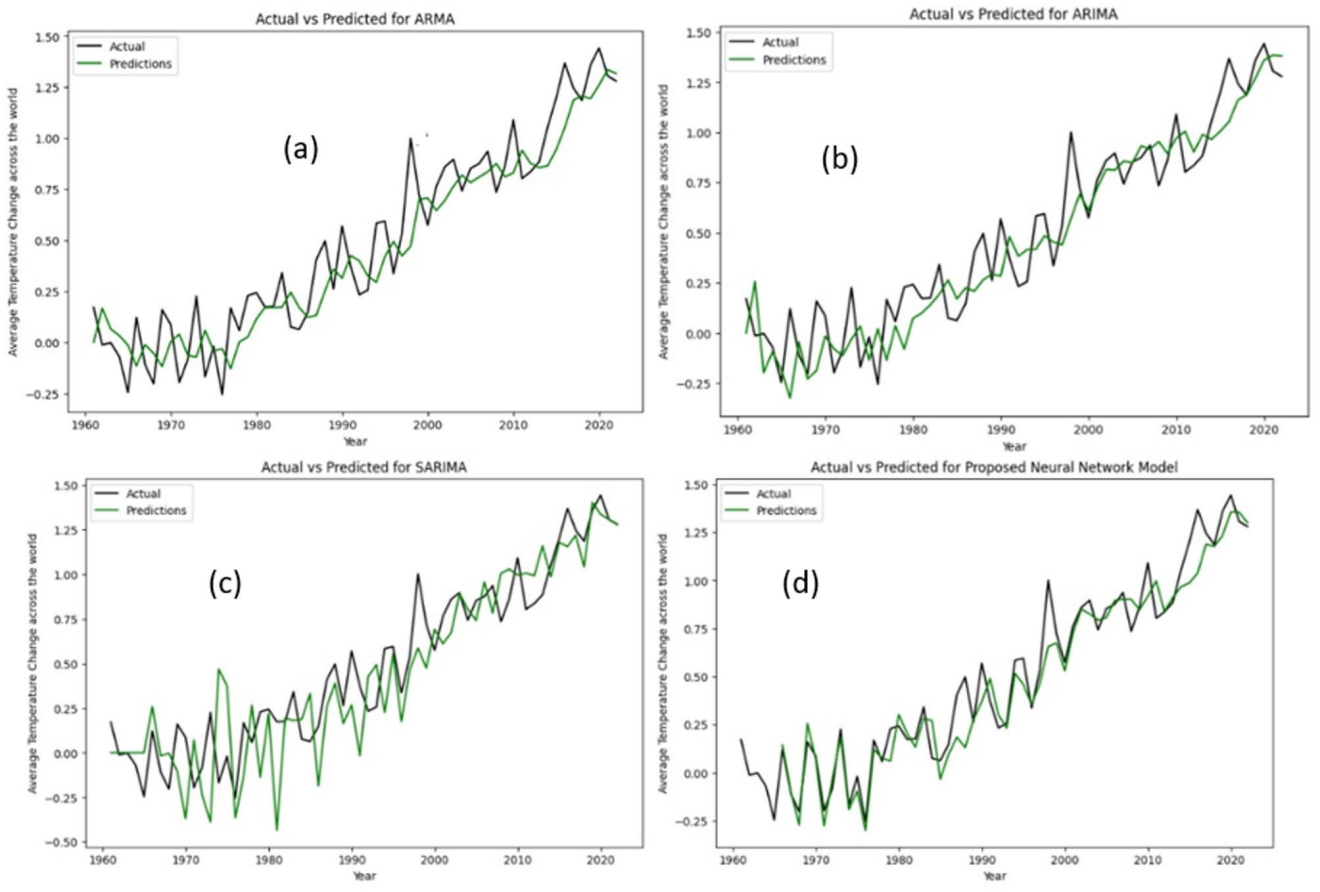
Actual data vs. predicted data for (**a**) ARMA, (**b**) ARIMA, (**c**) SARIMA and (**d**) Neural network model with Swish activation.

In this section, we explore the findings of our comparative analysis between the models, utilizing visual interpretations and comparison tables to convey the results and analysis effectively. First of all, we will visualize the actual vs. predicted line graphs of classical ML models followed by Neural Network models. Black line represents the actual data while the green line represents the predicted value by the respective model. Figure 3 shows the visual representation of the same for ARMA, ARIMA, SARIMA and Neural Network models with swish activation.

**Fig. 4:**
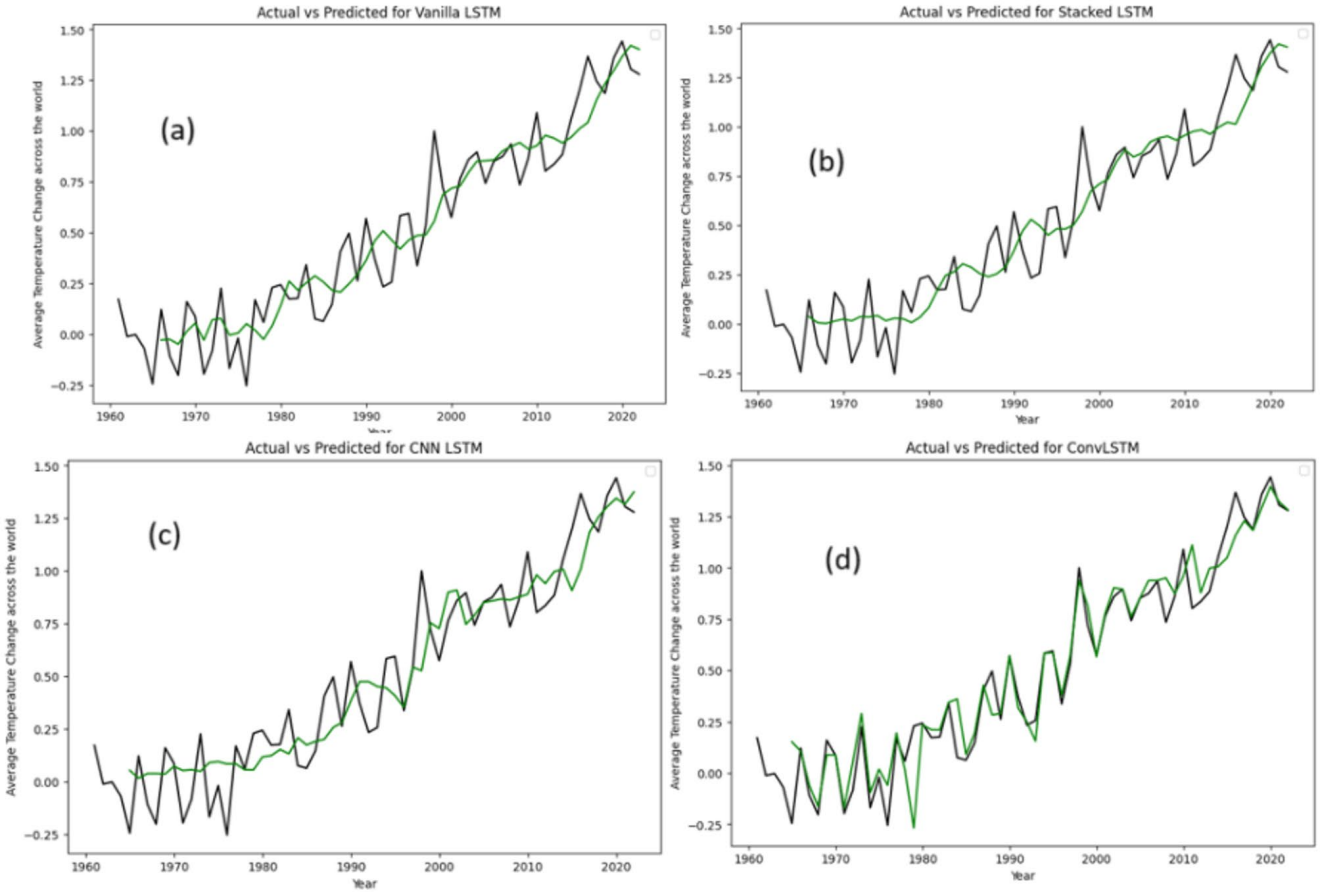
Actual data vs. prediction for (**a**) Vanilla LSTM, (**b**) Stacked LSTM, (**c**) CNN LSTM and (**d**) ConvLSTM model.

Subsequently, our analysis extends to the visualization of outcomes derived from an additional four neural network models. Figure 4 serves as a graphical depiction, presenting the results of the Vanilla LSTM, Stacked LSTM, CNN LSTM, and ConvLSTM models. Through these visual representations, we made comprehensive understanding of the performance and efficacy of each model.

**Fig. 5:**
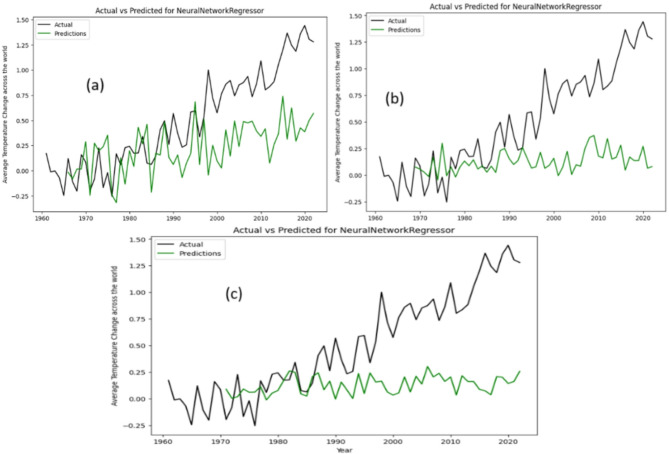
Actual data vs. prediction by NeuralNetworkRegressor model for (**a**) 5-Qubit, (**b**) 8-Qubit and (**c**) 10-Qubit.

Figure 5 provide insight into the Actual versus Predicted graphs for the quantum neural network (QNN) or NeuralNetworkRegressor model, leveraging 5, 8, and 10 qubits respectively. These visualizations offer a comparative analysis of the model’s performance across different qubit configurations. Notably, our observations indicate that while the QNN model demonstrates limited efficacy in time series forecasting, its potential shines through when applied to datasets characterized by greater stability over time. This nuanced understanding underscores the importance of considering the inherent dynamics and volatility of the dataset when evaluating the suitability of quantum computing approaches for predictive modeling tasks.

**Fig. 6:**
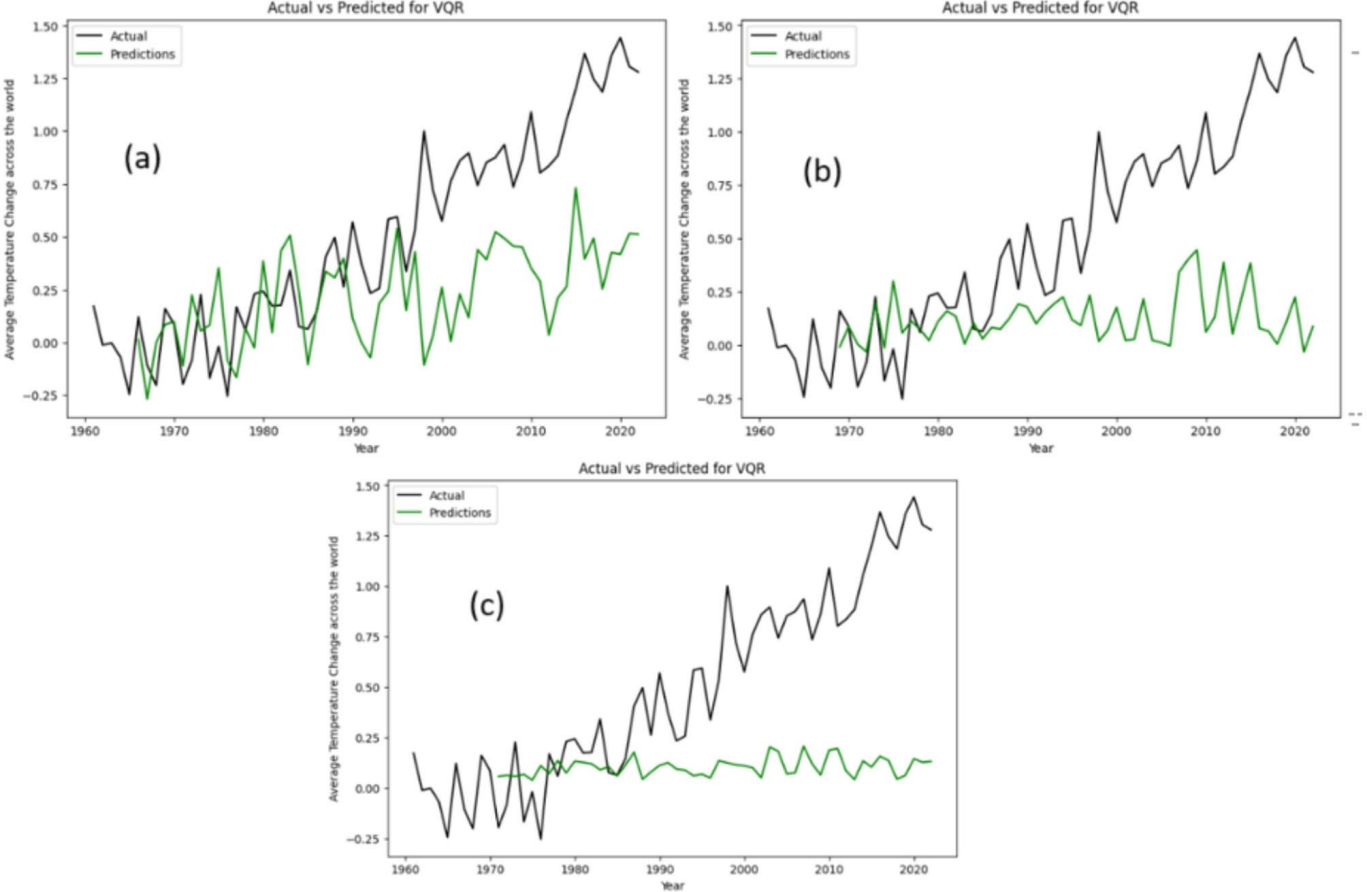
Actual data vs. prediction by VQR model for (**a**) 5-Qubit, (**b**) 8-Qubit and (**c**) 10-Qubit.

Likewise, Fig. 6 depict the actual versus predicted graphs for the variational quantum regression (VQR) model, utilizing 5, 8, and 10 qubits, respectively. These visualizations offer a comparative analysis of the VQR model’s performance across different qubit configurations. Our examination reveals striking similarities between the VQR and QNN models in terms of their predictive capabilities. Notably, both models exhibit promising results when applied to datasets characterized by greater stability over time. This shared attribute underscores a common trend in quantum computing methodologies, where the efficacy of predictive models is contingent upon the stability and consistency of the underlying data dynamics.

**Fig. 7:**
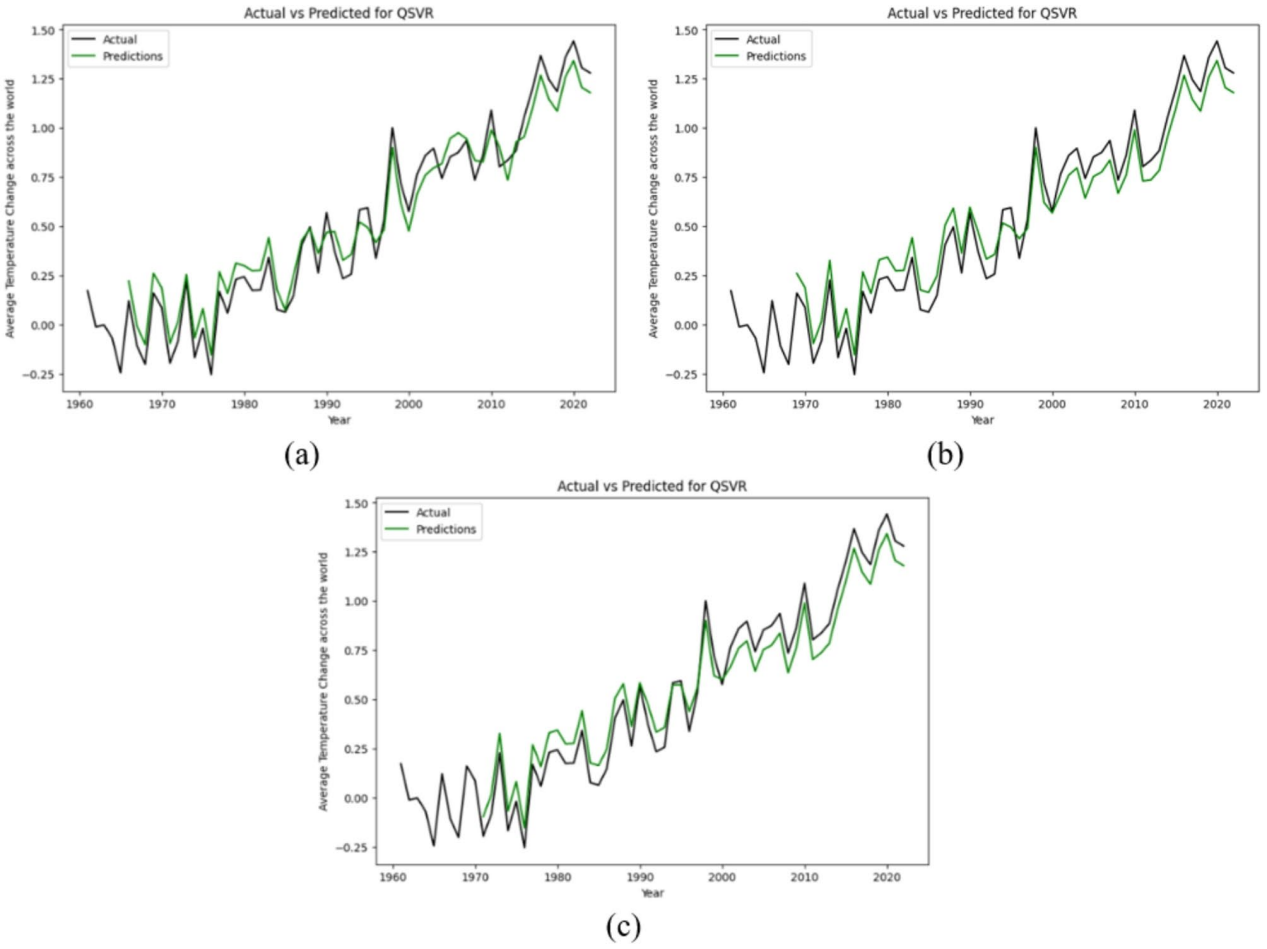
Actual data vs. prediction by QSVR model for (**a**) 5-Qubit, (**b**) 8-Qubit and (**c**) 10-Qubit.

Unlike the above two Quantum ML models, the QSVR model showcases a distinct approach to time series forecasting, exhibiting a remarkable precision that surpasses both classical ML and neural network models. Unlike many Quantum ML algorithms that primarily excel in linear data classification tasks, QSVR distinguishes itself by harnessing the power of Quantum Kernels. These kernels possess a unique adeptness in handling non-linear data structures, endowing QSVR with unparalleled capabilities to forecast diverse datasets with exceptional accuracy and ease. Figure 7 provides a visual representation of the actual versus predicted data generated by the QSVR model across 5, 8, and 10 qubit configurations. Notably, across all qubit configurations, QSVR consistently outperforms all other models implemented in this study. This striking superiority underscores the efficacy and versatility of QSVR in addressing the complexities inherent in time series forecasting, thus positioning it as a formidable contender in the realm of quantum-assisted predictive analytics.

**Fig. 8:**
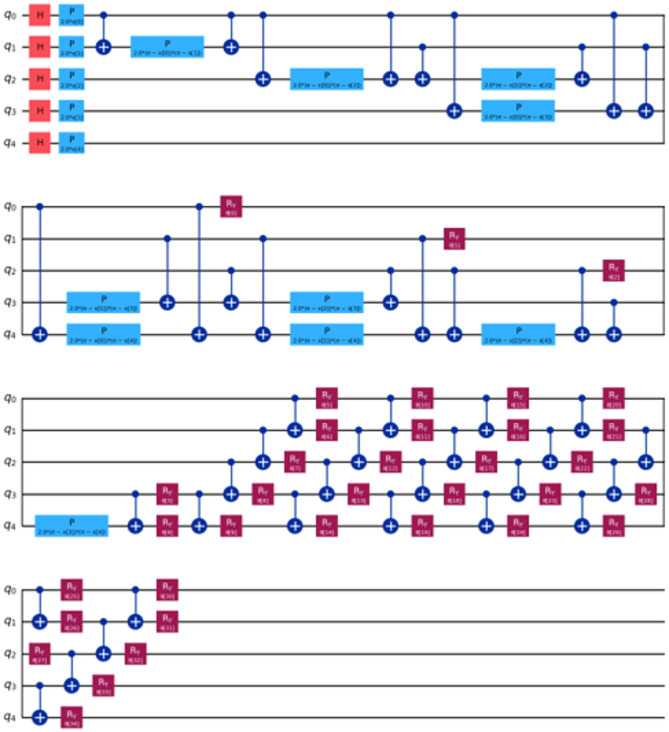
Quantum circuits used to train VQR 5-Qubit.

**Fig. 9:**
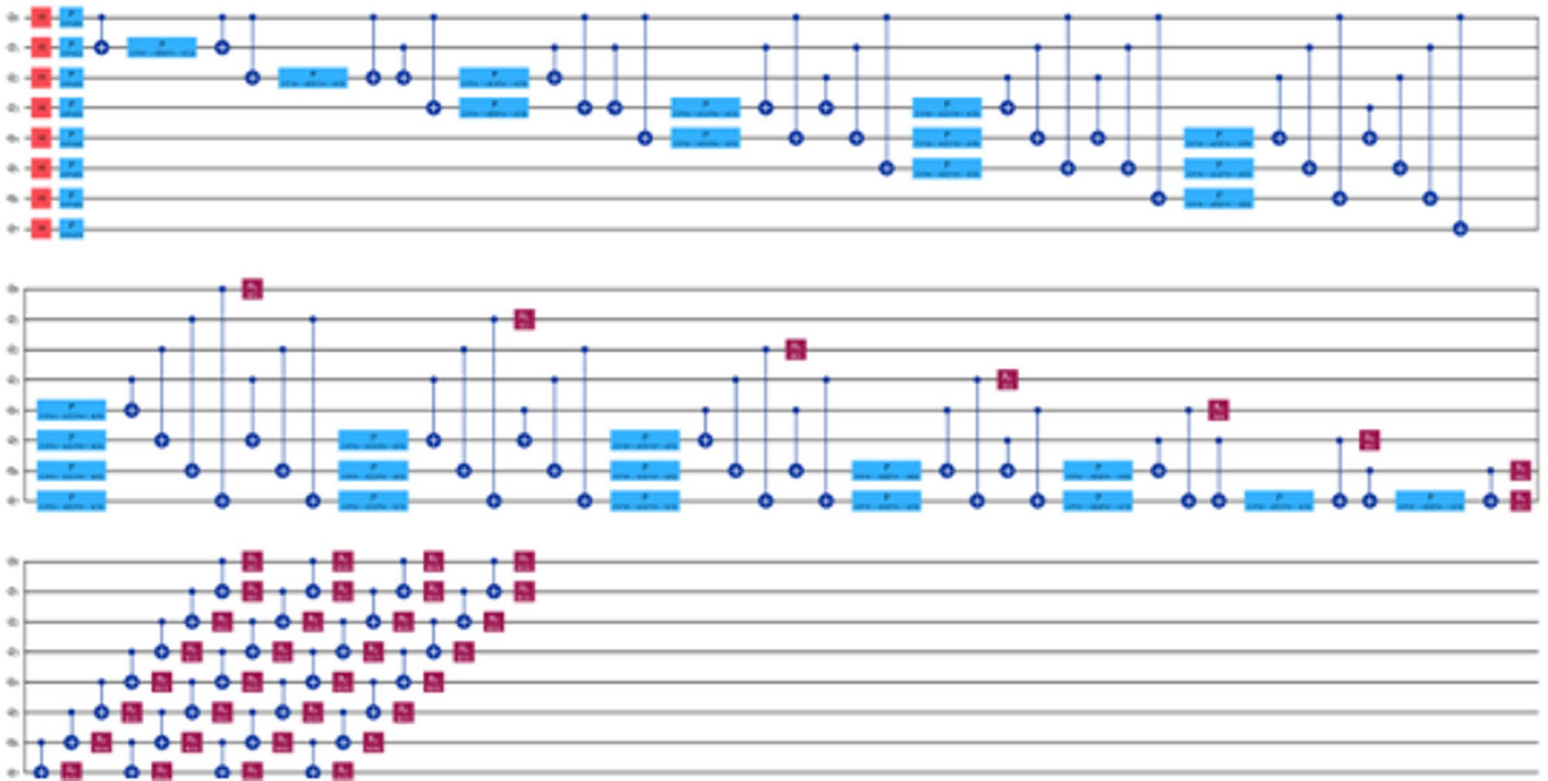
Quantum circuits used to train VQR 8-Qubit.

**Fig. 10:**
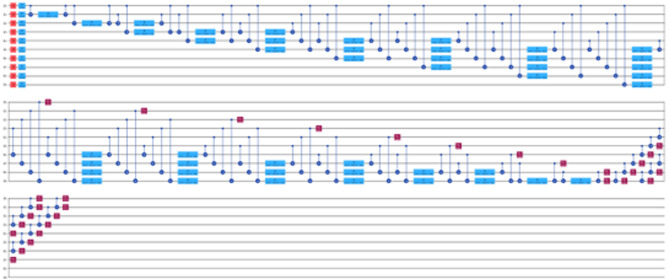
Quantum circuits used to train VQR 10-Qubit.

**Fig. 11:**
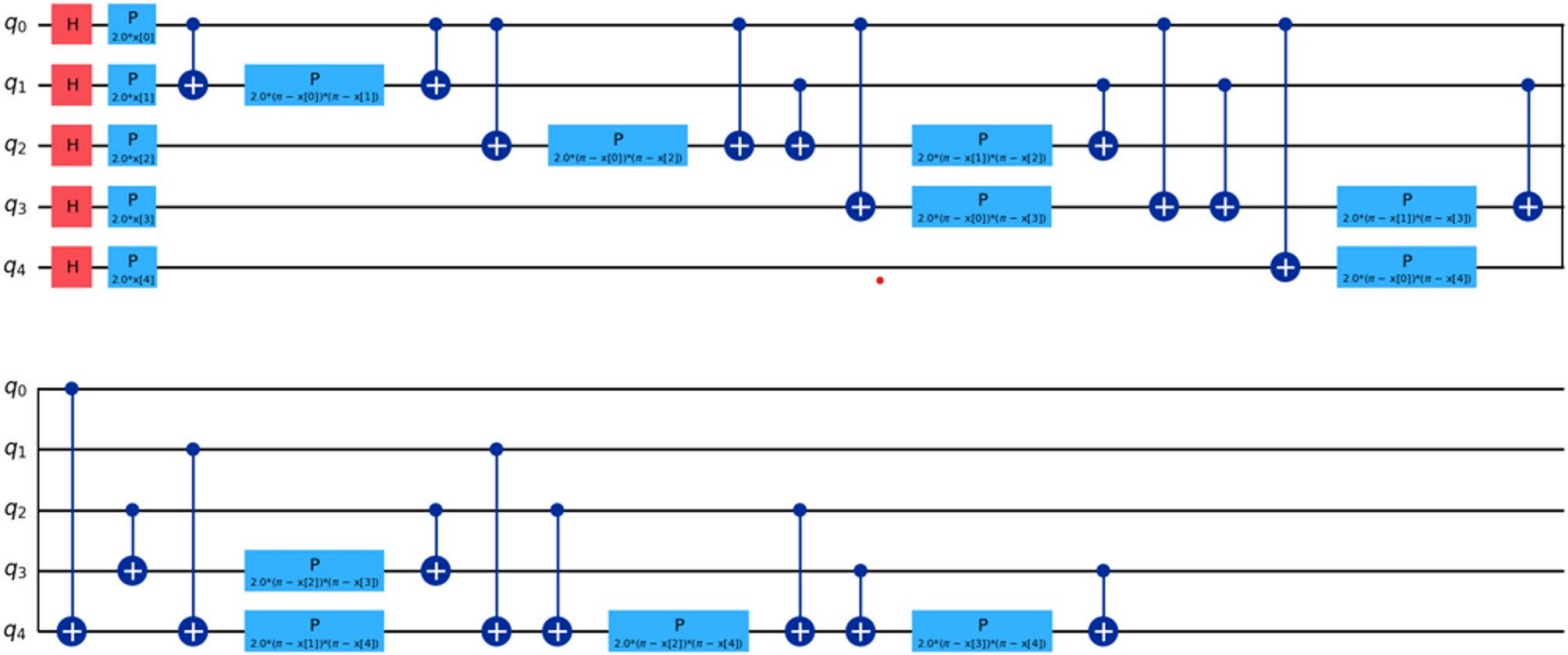
Feature maps used to train QSVR 5-Qubit system.

**Fig. 12:**
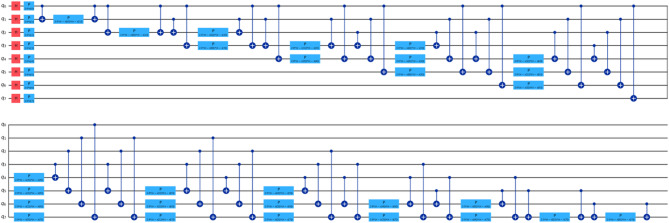
Feature maps used to train QSVR 8-Qubit system.

**Fig. 13:**
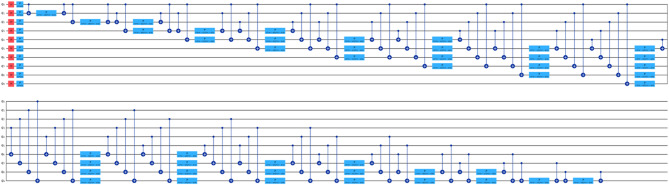
Feature maps used to train QSVR 10-Qubit system.

**Fig. 14:**
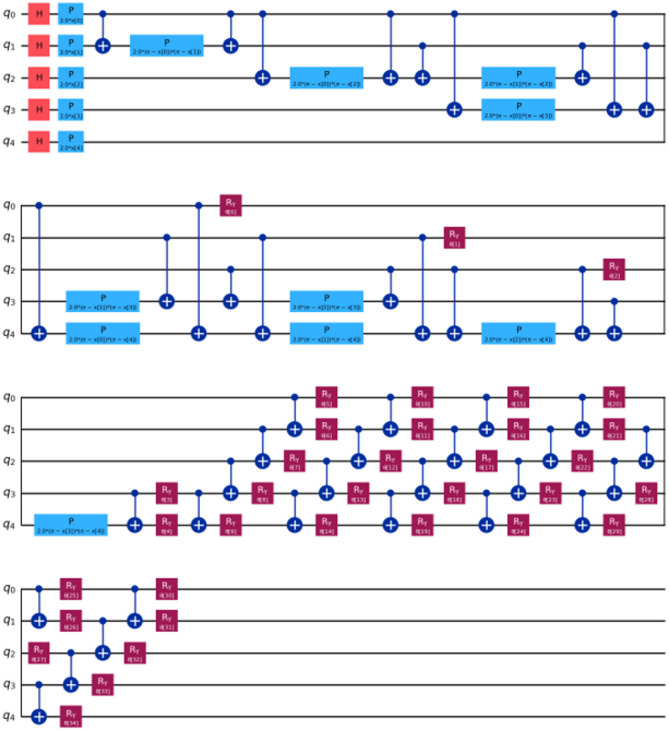
Quantum circuits used to train NeuralNetworkRegressor 5-Qubit.

For our comparative analysis, we have meticulously crafted a series of tables (Tables 7, 8, 9 and 10) to elucidate the performance metrics of Classical Machine Learning (ML) models, Neural Network Models, and Quantum ML algorithms. Each table provides a comprehensive overview of key evaluation metrics, including Root Mean Squared Error (RMSE), R^4^ Score, Mean Absolute Percentage Error (MAPE), and Time Elapsed in seconds, thereby facilitating a nuanced comparison across various algorithmic paradigms. Equations 3, 4 and 5 are used for calculation of these evaluation metrics. Table 7 meticulously presents the error rates and scores for Classical ML algorithms, shedding light on their predictive capabilities. Conversely, Table 8 delves into the performance metrics of Neural Network Models, providing insights into their efficacy in capturing complex patterns within the data. Tables 9 and 10 offer a detailed analysis of Quantum ML algorithms, with each table focusing on a specific qubit configuration—5-Qubit, 8-Qubit, and 10-Qubit, respectively. These tables meticulously showcase the error rates and scores for Quantum ML algorithms, simulated on classical computers and executed on quantum computers. The various kinds of quantum circuit models have been illustrated from (Figs. 8, 9, 10, 11, 12, 13 and 14).

**Table 7:** Results and time analysis for classical algorithms.

Classical machine learning algorithms				
Model	RMSE	*R*^2^ Score	MAPE	Time elapsed(s)
ARMA	0.1762	0.8620	1.2327	0.1090
ARIMA	0.1703	0.8710	2.3030	0.1470
SARIMA	0.2399	0.7441	1.1847	25.7510
Neural Network model with Swish activation	0.1197	0.9338	0.3248	1.4420
Vanilla LSTM	0.1539	0.8907	0.5160	2.2090
Stacked LSTM	0.1580	0.8848	0.5402	3.4680
CNN LSTM	0.1654	0.8776	0.5469	2.7780
ConvLSTM	0.1249	0.9302	0.3348	3.7880

**Table 8:** Results and time analysis for quantum algorithms (5-Qubit).

Quantum (5-Qubit)								
Simulation					IBM Lab			
Model	RMSE	*R*^2^ Score	MAPE	Time Elapsed	RMSE	*R*^2^ Score	MAPE	Time Elapsed(s)
QNN	0.4909	-0.1120	1.2678	204.8580	0.4121	0.2162	0.9724	125.6572
VQR	0.4954	-0.1324	1.1244	201.7796	0.4117	0.2178	0.8774	126.0125
**QSVR**	**0.0909**	**0.9618**	**0.4065**	**41.9790**	**0.0909**	**0.9618**	**0.4065**	**9.4226**

**Table 9:** Results and time analysis for quantum algorithms (8-Qubit).

Quantum (8-Qubit)								
Simulation					IBM Lab			
Model	RMSE	*R*^2^ Score	MAPE	Time Elapsed	RMSE	*R*^2^ Score	MAPE	Time elapsed(s)
QNN	0.6196	-0.8600	0.9976	347.9616	0.5422	-0.4216	0.9855	182.9478
VQR	0.6366	-0.9636	0.9931	307.0032	0.5807	-0.6335	0.9024	173.9459
**QSVR**	**0.0959**	**0.9555**	**0.4311**	**91.2959**	**0.0959**	**0.9555**	**0.4311**	**21.6689**

**Table 10:** Results and time analysis for quantum algorithms (10-Qubit).

Quantum (10-Qubit)								
Simulation					IBM Lab			
Model	RMSE	*R*^2^ Score	MAPE	Time Elapsed	RMSE	*R*^2^ Score	MAPE	Time Elapsed(s)
QNN	0.6823	-1.2575	0.8766	478.2502	0.6339	-0.9488	0.8436	109.6950
VQR	0.6475	-1.0330	0.8593	483.3490	0.6576	-1.0969	0.8610	108.7839
**QSVR**	**0.0949**	**0.9563**	**0.4092**	**136.3310**	**0.0949**	**0.9563**	**0.4092**	**48.1851**

## Conclusion

In conclusion, our analysis of quantum machine learning (QML) algorithms reveals a significant advantage in computational speed when executed on quantum computers compared to their simulation on classical computers. Among these algorithms, quantum support vector regression (QSVR) emerges as the standout performer in terms of both speed and accuracy. Our findings demonstrate that QSVR exhibits remarkable efficiency, with training times of 9.4, 21.6, and 48.1 s for systems utilizing 5, 8, and 10 qubits respectively. Moreover, it achieves impressive R^2^ scores of 0.9618, 0.9555, and 0.9563 for the respective qubit configurations. These results underscore its superiority as the premier QML algorithm for analyzing time-series data, surpassing even classical machine learning counterparts. Unlike many QML algorithms that primarily operate on linear data classification, QSVR stands out by leveraging Quantum Kernels. These kernels are adept at handling non-linear data, endowing QSVR with the capability to classify diverse datasets with remarkable ease and accuracy. Our study underscores the transformative potential of QML algorithms, particularly in handling complex datasets with nonlinear structures. As quantum computing continues to evolve, the practical applications of QML, exemplified by QSVR, hold promise for revolutionizing various fields reliant on data analysis and prediction. By harnessing the power of quantum computing, QML opens new avenues for addressing real-world challenges and advancing computational capabilities beyond the limitations of classical approaches.

## Data Availability

The datasets generated and/or analyzed during the current study are available in the FAOSTAT repository, Temperature change on land, https://www.fao.org/faostat/en/#data/ET and National Aeronautics and Space Administration National Aeronautics and Space Administration Goddard Institute for Space Studies, https://data.giss.nasa.gov/gistemp/.

## Code availability

The data that support the findings of this study are available from the corresponding author upon reasonable request.

## References

[1] Ding, C., Bao, T. Y. & Huang, H. L. Quantum-Inspired support vector machine. *IEEE Trans. Neural Netw. Learn. Syst.* **33**, 7210–7222 (2022). 34111003 10.1109/TNNLS.2021.3084467

[2] Sebestyén, V., Czvetkó, T. & Abonyi, J. The applicability of big data in climate change research: the importance of system of systems thinking. *Front. Environ. Sci.* 9. (2021).

[3] Abbas, A. et al. The power of quantum neural networks. *Nat. Comput. Sci.* **1**, 403–409 (2021). 38217237 10.1038/s43588-021-00084-1

[4] Abbass, K. et al. A review of the global climate change impacts, adaptation, and sustainable mitigation measures. *Environ. Sci. Pollut Res.* **29**, 42539–42559 (2022). 10.1007/s11356-022-19718-6 PMC8978769 35378646

[5] Adelomou, A. P., Ribe, E. G. & Cardona, X. V. Using the parameterized quantum circuit combined with variational-quantum-eigensolver (VQE) to create an intelligent social workers’ schedule problem solver. (2020).

[6] Dunjko, V. & Briegel, H. J. Machine learning & artificial intelligence in the quantum domain: a review of recent progress. *Rep. Prog Phys.* **81**, 074001 (2018). 29504942 10.1088/1361-6633/aab406

[7] Alexander, T. et al. Qiskit pulse: programming quantum computers through the cloud with pulses. *Quantum Sci. Technol.* **5**, 044006 (2020).

[8] Hanif, M., Sami, F., Hyder, M. & Ch, M. I. Hidden Markov model for time series prediction. *J. Asian Sci. Res.* **7**, 196–205 (2017).

[9] Altares-López, S., Ribeiro, A. & García-Ripoll, J. J. Automatic design of quantum feature maps. *Quantum Sci. Technol.* **6**, 045015 (2021).

[10] Khan, T. M. & Robles-Kelly, A. Machine learning: quantum vs classical. *IEEE Access.* **8**, 219275–219294 (2020).

[11] Azevedo, C., Ferreira, T. & Computing Time series forecasting with qubit neural networks. *Presented at the Proceedings of the 11th IASTED International Conference on Artificial Intelligence and Soft ASC 2007*. (2007).

[12] Banerjee, C., Mukherjee, T. & Pasiliao, E. An Empirical Study on Generalizations of the ReLU Activation Function. In *Proceedings of the 2019 ACM Southeast Conference, ACM SE ’19*. (Association for Computing Machinery, 2019).

[13] Designing Deep-Based Learning Flood Forecast Model With ConvLSTM Hybrid Algorithm |. IEEE Journals & Magazine | IEEE Xplore [WWW Document], n.d. URL.

[14] Botchkarev, A. Performance metrics (Error Measures) in machine learning regression, forecasting and prognostics: properties and typology. *Interdiscip J. Inf. Knowl. Manag*. **14**, 045–076 (2019).

[15] Lu, W., Li, J., Li, Y., Sun, A. & Wang, J. A CNN-LSTM-based model to forecast stock prices. *Complexity* e6622927 (2020).

[16] Chen, S. Y. C., Yoo, S. & Fang, Y. L. L. Quantum long short-term memory. (2020).

[17] Chicco, D., Warrens, M. J. & Jurman, G. The coefficient of determination R-squared is more informative than SMAPE, MAE, MAPE, MSE and RMSE in regression analysis evaluation. *PeerJ Comput. Sci.* **7**, e623. (2021). 10.7717/peerj-cs.623 PMC8279135 34307865

[18] Daskin, A. A walk through of time series analysis on quantum computers. (2022).

[19] Jadhav, A., Rasool, A. & Gyanchandani, M. Quantum machine learning: Scope for real-world problems. *Proc. Comput. Sci., International Conference on Machine Learning and Data Engineering* **218**, 2612–2625 (2023).

[20] Emmanoulopoulos, D. & Dimoska, S. Quantum machine learning in finance: Time series forecasting. (2022).

[21] Myers-Smith, I. H. et al. (2020). Complexity revealed in the greening of the Arctic. *Nat. Clim. Change* **10**, 106–117.

[22] Plevris, V., Solorzano, G., Bakas, N. P. & Ben Seghier, M. E. A. Investigation of performance metrics in regression analysis and machine learning-based prediction models. 8. *Eur. Commun. Computat. Methods Appl. Sci.* (2022).

[23] Sherstinsky, A. Fundamentals of recurrent neural network (RNN) and long Short-Term memory (LSTM) network. *Phys. Nonlinear Phenom.* **404**, 132306 (2020).

[24] Inoue, T. et al. Clustering method for time-series images using quantum-inspired computing technology. (2023).

[25] Mücke, S., Heese, R., Müller, S., Wolter, M. & Piatkowski, N. Feature selection on quantum computers. *Quantum Mach. Intell.* **5**, 11 (2023).

[26] Anomalies, U. *An End-to-End Seasonal-Trend Decomposition Approach for Time Series Anomaly Detection | IEEE Conference Publication | IEEE Xplore [WWW Document]*, n.d.

[27] Dubey, S. R., Singh, S. K. & Chaudhuri, B. B. Activation functions in deep learning: A comprehensive survey and benchmark. *Neurocomputing* **503**, 92–108 (2022).

[28] Pira, L. & Ferrie, C. An invitation to distributed quantum neural networks. *Quantum Mach. Intell.* **5**, 23 (2023).

[29] Wille, R., Van Meter, R. & Naveh, Y. IBM’s Qiskit tool chain: Working with and developing for real quantum computers. In *2019 Design, Automation & Test in Europe Conference & Exhibition (DATE). Presented at the 2019 Design, Automation & Test in Europe Conference & Exhibition (DATE)* pp. 1234–1240. (2019).

[30] Jia, Z. A. et al. Quantum neural network states: A brief review of methods and applications. *Adv. Quantum Technol.* **2**, 1800077 (2019).

[31] Kingma, D. P. & Ba, J. Adam: A method for stochastic optimization. (2017).

[32] Martonosi, M. & Roetteler, M. *Next Steps in Quantum Computing* (Computer Science’s Role, 2019).

[33] Rivera-Ruiz, M. A., Mendez-Vazquez, A. & López-Romero, J. M. Time series forecasting with quantum machine learning architectures. In *Advances in Computational Intelligence* (eds Pichardo Lagunas, O. et al.) 66–82 (Springer Nature Switzerland, 2022).

[34] Mastriani, M. Quantum spectral analysis: frequency in time, with applications to signal and image processing. (2021).

[35] Gao, L. Z., Lu, C. Y., Guo, G. D., Zhang, X. & Lin, S. Quantum K-nearest neighbors classification algorithm based on Mahalanobis distance. *Front. Phys.* 10. (2022).

[36] Suzuki, T., Hasebe, T. & Miyazaki, T. Quantum support vector machines for classification and regression on a trapped-ion quantum computer. (2023).

[37] Schuld, M. Supervised quantum machine learning models are kernel methods. (2021).

[38] Zhou, X., Yu, J., Tan, J. & Jiang, T. Quantum kernel estimation-based quantum support vector regression. *Quantum Inf. Process.* **23**, 29 (2024).

[39] Pulicharla, M. R. Hybrid Quantum-Classical machine learning models: powering the future of AI. *J. Sci. Technol.* **4**, 40–65 (2023).

[40] TimeDistributed-CNN-LSTM A Hybrid Approach Combining CNN and LSTM to Classify Brain Tumor on 3D MRI Scans Performing Ablation Study | IEEE Journals & Magazine | IEEE Xplore [WWW Document], n.d.

[41] Tahmasebi, P., Kamrava, S., Bai, T. & Sahimi, M. Machine learning in geo- and environmental sciences: from small to large scale. *Adv. Water Resour.* **142**, 103619 (2020).

[42] Mercioni, M. A. & Holban, S. P-Swish: Activation function with learnable parameters based on Swish activation function in deep learning. In *2020 International Symposium on Electronics and Telecommunications (ISETC). Presented at the 2020 International Symposium on Electronics and Telecommunications (ISETC)*. 1–4. (2020).

[43] Yu, L., Qu, J., Gao, F. & Tian, Y. A novel hierarchical algorithm for bearing fault diagnosis based on stacked LSTM. *Shock Vib.* e2756284. (2019).

[44] Moreira, C. & Wichert, A. Quantum-Like bayesian networks for modeling decision making. *Front. Psychol.* **7**. (2016). 10.3389/fpsyg.2016.00011 PMC4726808 26858669

[45] Wang, C. C. J. & Bennink, R. S. Variational quantum regression algorithm with encoded data structure. (2024).

[46] Thompson, N. C., Greenewald, K., Lee, K. & Manso, G. F. The computational limits of deep learning. (2022).

[47] Yu, K., Lin, S. & Guo, G. D. Quantum dimensionality reduction by linear discriminant analysis. *Phys. Stat. Mech. Its Appl.* **614**, 128554 (2023).

